# Measurement of Neurovascular Coupling in Neonates

**DOI:** 10.3389/fphys.2019.00065

**Published:** 2019-02-18

**Authors:** Dries Hendrikx, Anne Smits, Mario Lavanga, Ofelie De Wel, Liesbeth Thewissen, Katrien Jansen, Alexander Caicedo, Sabine Van Huffel, Gunnar Naulaers

**Affiliations:** ^1^Department of Electrical Engineering, KU Leuven, Leuven, Belgium; ^2^imec, Leuven, Belgium; ^3^Department of Development and Regeneration, KU Leuven, Leuven, Belgium; ^4^Neonatal Intensive Care Unit, University Hospitals Leuven, Leuven, Belgium; ^5^Child Neurology, University Hospitals Leuven, Leuven, Belgium; ^6^Facultad de Ciencias Naturales y Matemáticas, Universidad del Rosario, Bogotá, Colombia

**Keywords:** neurovascular coupling, neonates, cerebral blood flow, EEG, NIRS, graph theory

## Abstract

Neurovascular coupling refers to the mechanism that links the transient neural activity to the subsequent change in cerebral blood flow, which is regulated by both chemical signals and mechanical effects. Recent studies suggest that neurovascular coupling in neonates and preterm born infants is different compared to adults. The hemodynamic response after a stimulus is later and less pronounced and the stimulus might even result in a negative (hypoxic) signal. In addition, studies both in animals and neonates confirm the presence of a short hypoxic period after a stimulus in preterm infants. In clinical practice, different methodologies exist to study neurovascular coupling. The combination of functional magnetic resonance imaging or functional near-infrared spectroscopy (brain hemodynamics) with EEG (brain function) is most commonly used in neonates. Especially near-infrared spectroscopy is of interest, since it is a non-invasive method that can be integrated easily in clinical care and is able to provide results concerning longer periods of time. Therefore, near-infrared spectroscopy can be used to develop a continuous non-invasive measurement system, that could be used to study neonates in different clinical settings, or neonates with different pathologies. The main challenge for the development of a continuous marker for neurovascular coupling is how the coupling between the signals can be described. In practice, a wide range of signal interaction measures exist. Moreover, biomedical signals often operate on different time scales. In a more general setting, other variables also have to be taken into account, such as oxygen saturation, carbon dioxide and blood pressure in order to describe neurovascular coupling in a concise manner. Recently, new mathematical techniques were developed to give an answer to these questions. This review discusses these recent developments.

## Introduction

**FIGURE 1:**
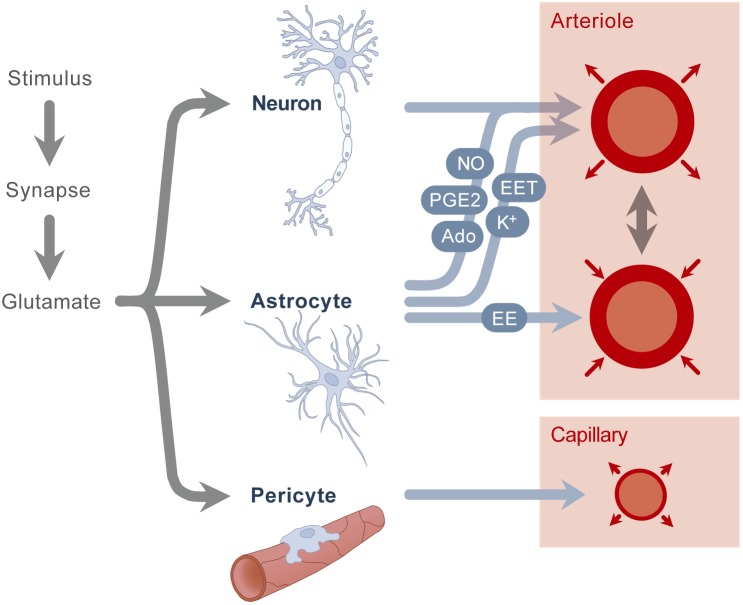
A stimulus causes the secretion of glutamate. Glutamate stimulates neurons and astrocytes, which results in the secretion of nitric oxide (NO), potassium (K^+^), adenosine (Ado), epoxyeicosatrienoic acids (EET) and prostaglandins (PGE2), which in turn results in arteriolar vasodilation. Astrocytes also secrete arachidonic acid (AA), which causes vasoconstriction. A second mechanism is the stimulation of pericytes, resulting in capillary vasodilation.

Neurovascular coupling refers to the regulation mechanism that links the transient neural activity to the subsequent change in cerebral blood flow. The first reports on this regulation mechanism date from more than 100 years ago (Donders, 1851). Neurovascular coupling can be studied from different points of view, including a macroscopic and microscopic perspective. At the macroscopic level, the extremely high vascularization and tight regulation of the cerebral blood flow provides the brain with adequate blood flow for a given metabolic demand. There is a close temporal and regional link between neuronal activity and cerebral blood flow, brain regions with high activity receive an increased amount of blood flow. At the microscopic level, the neurovascular unit is comprised of the vascular smooth muscle, the neuron and the astrocyte glial cell. Glutamate is released upon neuronal activation, which causes both neurons and astrocytes to transmit signals in order to regulate cerebral blood flow. Although it was assumed for a long time that these signals were only associated with a vasodilatory effect, recent studies show a more complex balance of vasodilation and vasoconstriction, in which both chemical and mechanical effects play an important role. Known chemical signals include prostaglandins, nitric oxide and adenosine, secreted by both neurons and astrocytes, which cause a vasodilation of the smooth muscle cell (Figure 1). In addition, astrocytes also secrete other vasodilators like potassium and epoxyeicosatrienoic acids. Besides these vasodilators, astrocytes are also known to secrete arachidonic acid, which has a vasoconstrictive effect. All these mediators have a direct impact on the smooth muscle of the arterioles and therefore control cerebral blood flow in a direct way. In addition to these mechanisms present in the neurovascular unit, a second mechanism was recently described by which also pericytes around the capillaries cause vasodilation, probably also playing a role in the local flow distribution. For further description of these mechanisms, we refer to recent reviews (Huneau et al., 2015; Phillips et al., 2016). As a concluding remark, it is also important to mention that most of the studies on neurovascular coupling are done in the adult population; currently, we do not know whether these mechanisms are present in preterm neonates, and if they work in the same way as described in adults.

In general, there are two main categories of studies to describe and investigate neurovascular coupling. First of all, there are *spatiotemporal* studies, which link changes in local blood flow to an artificially applied stimulus. Second, *general* studies are available that investigate resting-state neurovascular coupling using spontaneous electrical activity of the brain.

## Spatiotemporal Studies

In order to test neurovascular coupling hypotheses in adults, spatiotemporal studies are standard of practice. In such studies, a standardized motor, visual, auditive, or cognitive task is given and simultaneously, the flow and/or activity in the brain is measured. There are different recommendations regarding optimal stimulus and assessment methods for adult research (Huneau et al., 2015). The most widely used stimulus in adults is finger tapping, which is known to cause an increase in blood flow at the contralateral motor cortex (Dettmers et al., 1996). In (preterm) neonates, spatiotemporal studies are also of interest, but they are inherently more difficult to perform. The majority the spatiotemporal studies in neonates makes use of visual stimuli, which includes the projection of checkerboard patterns on LCD displays (Meek et al., 1998; Liao et al., 2010), or flashing LEDs (Karen et al., 2008). The latter has the advantage that the neonates can be kept asleep throughout the measurements. Other less frequently used stimuli in preterm neonates include auditory (Sakatani et al., 1999; Zaramella et al., 2001) and somatosensory stimuli (Erberich et al., 2006; Arichi et al., 2010, 2012). To the best of our knowledge, guidelines regarding optimal stimulus selection for newborns are not available.

The classical response to the stimulus described in adults is a sudden increase in cerebral blood flow and cerebral oxygenation with a secondary, less pronounced decrease (Heekeren et al., 1997). This type of response is typically called ‘functional hyperemia,’ and is generally referred to as a *positive* response. A *negative* response, on the other hand, occurs when the increase in blood flow to the brain is insufficient to meet the metabolic demand. In this setting, however, the relation of functional hyperemia to neuronal metabolisms is unclear (Hillman, 2014). Since functional hyperemia is observed in hyperoxic, hypoglycemic and hyperglycemic states, it is clear that the blood flow increases are not simply triggered by local sensing of depleted nutrients (Powers et al., 1996; Wolf et al., 1997; Lindauer et al., 2010). The relative delay in the peak of increased blood flow further confirms that neurons do not rely upon functional hyperemia to meet their initial needs for increased oxygen and glucose, since neuronal firing may have ended prior to measurable changes in blood flow (Hillman, 2014).

In practice, different techniques are used to test neurovascular coupling in humans. In *spatiotemporal* studies, functional magnetic resonance imaging (fMRI) and functional near-infrared spectroscopy (fNIRS) are the most frequently used methods. The fMRI studies investigate the response of the blood oxygen level dependent (BOLD) signal. According to the definition, fMRI and fNIRS studies only measure blood flow changes and thus not neural activity. However, since the change in blood flow occurs mainly at the part of the brain responsible for the imposed activity (verbal, motor, visual), the measured response can be a good surrogate measurement for neurovascular coupling. In addition to the sign of the underlying mechanism (*positive* versus *negative* response), fMRI studies are also used to determine the location of brain activity after a specific stimulus. In healthy humans, cognitive, verbal, and motor tasks will on average lead to a 10–20% increase in cerebral blood flow in the posterior cerebral artery and a 5–8% increase in the middle cerebral artery (Huneau et al., 2015). In fNIRS studies, a positive response is comprised of an increase in oxyhemoglobin and total hemoglobin, together with a slight decrease in deoxygenated hemoglobin, which overall leads to an increase in tissue oxygenation. In adults, impaired neurovascular coupling has been described in different pathological conditions like stroke, hypertension, hypotension, autonomic dysfunction, Alzheimer’s disease, diabetes, and traumatic brain injury (Duschek and Schandry, 2004; Girouard and Iadecola, 2006; Azevedo et al., 2011; Vetri et al., 2012; Phillips et al., 2014; Jang et al., 2017). In addition, also smoking and healthy aging are associated with a negative effect on neurovascular coupling (Boms et al., 2010; Tarantini et al., 2017; Nowak-Fluck et al., 2018).

Besides the use of fMRI and fNIRS, other techniques can also be used to carry out spatiotemporal studies. These techniques are however less frequently used – and, to the best of our knowledge – not yet employed in studies on neurovascular coupling in preterm neonates. fMRI BOLD imaging depends on specific coupling relations between cerebral metabolic rate of oxygen, cerebral blood flow and cerebral blood volume, which are not unambiguously described in preterm neonates to date (Muramoto et al., 2002; Kusaka et al., 2004). Furthermore, these relations are altered in case of brain injury (Lin et al., 2016). Perfusion-MRI techniques, such as arterial spin labeling (ASL) MRI, can be used to overcome these limits (Tortora et al., 2017). In addition, oxygen based techniques such as BOLD fMRI and fNIRS only perform indirect measures of functional hyperemia (Hyder, 2009). More precise, direct measurements of cerebral blood flow can be obtained using laser Doppler flowmetry (Dyson et al., 2014) or laser speckle imaging (Farraro et al., 2016). A disadvantage of these techniques is the sensitivity of the probe, which results in signals that are prone to artifacts, and which impedes continuous measurements – typically, laser imaging measurements are taken at discrete points in time. A new promising technique is functional ultrasound imaging, where fast ultrasound sonography is coupled with EEG (Demene et al., 2017).

Recent studies suggest that neurovascular coupling in neonates and preterm born infants can differ compared to adults. This is typically attributed to the fact that many of the components involved in actuating and propagating the hemodynamic response are still in further development, including perivascular cells such as astrocytes and pericytes (Kozberg and Hillman, 2016a). Neural and vascular networks develop, expand and are then selectively pruned over the first year of human life. In addition, the metabolic demands of the newborn brain are still evolving and are vastly different compared to the adult brain. On the first day after birth, very low values of brain oxygenation are observed, in combination with high values of cerebral oxygen extraction (Brew et al., 2014). Brain oxygenation increases during the first days of life, while cerebral oxygen extraction decreases. In extremely preterm infants, there is no correlation between cerebral blood flow and spontaneous changes in the cerebral metabolic rate of oxygen during the first 2 days after birth (Kissack et al., 2005; Wong et al., 2009). Instead, changes in cerebral oxygen extraction rather than cerebral blood flow meet changes in oxygen requirements arising from variations of the cerebral metabolic rate of oxygen. The vast differences in both neural and vascular network structure, as well as substantially different metabolic needs of the preterm brain are highly likely to affect early postnatal neurovascular coupling.

The differences in neurovascular coupling hypotheses in neonates versus adults are confirmed in fMRI studies, which are discussed in this paragraph, and in fNIRS studies, which are summarized in the next paragraph. Heep et al. (2009) described a negative response in preterm infants after stimulation, which was also confirmed by others (Yamada et al., 1997; Born et al., 1998; Erberich et al., 2006). Arichi et al. (2012) found a positive BOLD response in adults, term infants and preterm infants. Furthermore, they identified a systematic maturational trend in terms of decreasing time-to-peak and increasing positive peak amplitude. These findings suggest that in young infants the increase in cerebral oxygen consumption may be relatively greater than the corresponding increase in cerebral blood flow during functional activation. The age-dependency of the neurovascular coupling was also confirmed in a rodent model by Kozberg et al. (2013). The development of the neurovascular coupling alongside neuronal development in the postnatal brain suggests that in the developmental period the brain may be experiencing differences in energy supply and demand dynamics compared to the adult brain (Kozberg and Hillman, 2016a).

Another approach to study the neurovascular coupling is by means of fNIRS. Again, the results and conclusions of the different studies available in literature are not unambiguous. Liao et al. (2010) described a normal hyperemic signal using NIRS in the visual cortex after visual stimulation in a cohort of term neonates in the first weeks of life. Furthermore, no simultaneous reaction was observed at the motor cortex, indicating that the observed response was a local reaction. On the other hand, however, an increase in brain deoxygenation was observed in awake infants in another study (Meek et al., 1998). Verriotis et al. (2016) described the response on noxious (heel lance prick) and innoxious stimuli (tactile cutaneous stimulation) in 30 term infants. In general, noxious stimulation was found to elicit a more pronounced hemodynamic response than innoxious stimulation. However, the hemodynamic pattern after stimulation was observed to be characterized by pronounced inter-patient differences, which might suggest that increased oxygen consumption does not always lead to regional overperfusion, likely due to the immature vascular regulation or to greater metabolic demands of neurotransmission in unmyelinated white matter.

In addition to the fNIRS studies on neonates, other authors have used this technique on animals. Nakamura et al. (2017) studied the neurovascular coupling in newborn lambs. They found subjects with a positive response, i.e., an increase in oxygenated hemoglobin, but also subjects with a negative response after a motor stimulation. Furthermore, blood pressure at the start of the investigation did not seem to have any influence, but the negative response became positive in hypercapnia (Nakamura et al., 2017). Kozberg et al. (2016) studied the neurovascular coupling in rats of different ages. They described a less positive response in rats of postnatal age P10–P13, and even a negative response in rats of postnatal age P7–P8. In order to compare these results to the human population, it is common to assume that the newborn full-term and 1 year old human brain are developmentally equivalent to the postnatal days 7 and 14 in rats, respectively, based on the codevelopment of factors such as eye opening and myelination (Quinn, 2005).

In addition to the uncertainty regarding the sign of the hemodynamic response (*positive* versus *negative*) in neonates, more questions arise when we focus on specific pathologic conditions like bronchopulmonary dysplasia, congenital heart disease and pulmonary hypertension, where a lower oxygen saturation is present (Rosengarten et al., 2006). Furthermore, the effect of anesthetic and sedative drugs, which are frequently used in fMRI studies, needs to be investigated (Franceschini et al., 2010). From a broader perspective, also the effect of other drugs, such as anti-epileptic drugs on neurovascular coupling in neonates is not properly defined to date (Schwartz, 2007). Since an elaborate discussion on these topics is out of the scope of this text, we will not go into further detail.

It is important to mention that studies both in animals and neonates confirm the presence of a short hypoxic period in the brain after a stimulus in preterm infants. These short hypoxic moments are hypothesized to cause an increase in vascular endothelial growth factor and oligodendrocyte-encoded hypoxia-inducible factor function, resulting in new angiogenesis and neurogenesis in the brain (Kozberg and Hillman, 2016b). This hypothesis could explain the mechanisms of the positive effect of sensitive stimulation and the negative effect of overstimulation in newborns (Kozberg and Hillman, 2016a).

Despite the fact that the fMRI and fNIRS studies listed above aim to investigate neurovascular coupling, they actually only describe the hemodynamic changes observed in the brain after applying a stimulus. These studies can be used to describe these hemodynamic changes after a particular stimulus and how these hemodynamic patterns change with age, from preterm neonates to adults. Precise studies on neurovascular coupling are based on concomitant measurements of brain hemodynamics and brain function (electrical activity of the brain). This can be done by combining EEG measurements with fMRI measurements (Mullinger and Bowtell, 2011), with fNIRS measurements (Shin et al., 2018) or with positron emission tomography measurements (Juhász et al., 2000), although such studies are challenging from a technical point of view due to for example electrode heating (Vanhatalo et al., 2014) and the presence of numerous artifacts (Galderisi et al., 2016; Abrue et al., 2018). Therefore, such studies are very scarce, especially in (preterm) neonates. Singh et al. (2014) explored the use of concomitant multichannel EEG-fNIRS measurements during epileptic seizures in neonates and found a hemodynamic response to seizure activity which consists of an initial increase in cortical blood volume prior to a large and extended decrease typically lasting several minutes.

## General Studies

Another approach to investigate the neurovascular coupling is to study the relation between the general metabolism of the brain and cerebral blood flow. Obviously, EEG is the most commonly used non-invasive method to assess the electrical activity of the brain, although it remains limited to the cortical activity. Cerebral blood flow can be measured at the bedside in a non-invasive way using transcranial Doppler ultrasound or near-infrared spectroscopy (NIRS). Therefore, these two modalities are generally used in *general* studies on neurovascular coupling. Both technologies are safe, relatively cheap and easy to use, however, ultrasound imaging has the disadvantage of being investigator dependent.

Roche-Labarbe et al. (2007) used NIRS in combination with EEG and found a decrease in oxygenation during bursts (high EEG activity in very preterm infants), followed by an important overflow (increase in cerebral oxygenation), suggesting a neurovascular signal that differs from the pattern commonly observed in adults. Tataranno et al. (2015) investigated functional brain activity and found an increase in oxygen extraction in preterm infants with increased electro-cerebral activity. In another study, Mahmoudzadeh et al. (2018) compared the neurovascular coupling in preterm neonates with versus without intraventricular hemorrhage. They reported that in neonates with brain injury the cerebral vascular network was unable to compensate for the increased metabolism resulting from neuronal activation in response to external stimulation. Pfurtscheller et al. (2008) investigated the behavior of the heart rate (HR) variability during the preterm EEG transients and found that EEG burst are associated with increases in HR. In addition, this positive variation of HR was found to disappear over age with the emergence of a continuous EEG trace.

The most recent advancements in signal processing allow an investigation of the neurovascular coupling in a non-invasive way focusing on “resting-state” conditions. In such a setting, the clinical goal is to develop a non-invasive and continuous measurement of the neurovascular coupling, which can be used as bedside monitoring without evoking a potential, i.e., inducing an artificial response via a stimulus. In the past decades, one of the most commonly studied neuronal couplings has been the scalp EEG connectivity, for which a large variety of methodologies has been developed (Pereda et al., 2005). In general, the methods can be classified in *functional methods* on the one hand, if only statistical correlations among the time series are taken into account, versus *effective methods* on the other hand, if the directionality of the coupling is considered as well (Friston, 2011). An overview of these methodologies is presented in Table 1.

**Table 1:** Overview of the signal processing techniques used to assess neurovascular coupling by integrating NIRS and EEG measurements.

	Linear techniques	Non-linear techniques
Functional methods	► Time domain • Correlation • Time delay stability ► Frequency domain • Coherency ► Time-frequency domain • Wavelet coherency	► Information theory • Mutual information ► Dynamic time warping ► Phase locking value
Effective methods	► Transfer function ► Granger causality	► Information theory • Transfer entropy ► Evolutionary map approach (directionality index)

Based on the EEG connectivity literature, further coupling methodologies have been developed to assess the interaction among signals with different origins or sources. These methodologies can consequently be used to assess regulation mechanisms like neurovascular coupling. Clinical examples on neurovascular coupling are the studies of Pfurtscheller et al. (2008) and Roche-Labarbe et al. (2008). Although these studies investigate the interaction of signals of different nature, these studies do not assess the concise nature of their interaction. The main difficulty to tackle in studies on multimodal integration is the fact that the different time series operate on different time scale, while simultaneously interacting with each other with a certain linear or non-linear degree.

In order to overcome this limitation, the new field of Network Physiology has the scope to describe how the time series of multiple origins interact, either linearly or non-linearly at a specific scale, in a graph or lattice structure (Bashan et al., 2012; Bartsch et al., 2015). Based on a Network Physiology framework, this review will explain new mathematical models to assess the neurovascular coupling, with a specific focus on:

(1) The necessary preprocessing steps that are required to match the temporal scales at which neuronal and vascular activities interact,
(2) The variety of methods to assess the coupling itself, which includes linear and non-linear approaches,
(3) The graph theory approach to describe the multiple simultaneous interactions and manage them in a statistically compact way.

### Preprocessing Steps

In order to measure the neurovascular coupling in a correct and robust way, it is very important that the dynamics of the various signals are represented and matched in a careful way. In general, biomedical signals arise from a variety of sources, including movements, breathing, electrical activity of neurons (EEG) and optical absorption of light (NIRS), among others (Clemenson and Lancaster, 2016). Due to the different sources, biomedical time series are typically associated with different time scales of operation. An EEG signal, for example, changes rapidly due to the (de)synchronization of numerous neurons, while a NIRS signal only changes very slowly, due to the fact that the hemodynamic effects that it captures work on a slow time scale. When one wants to compute the interaction between two signals, it is important to define on which time scale the coupling is ought to be computed (the signals are interacting), and the signals have to be matched correspondingly. Although biological systems are extremely complex, it is possible to decompose their macroscopic behavior using mathematical techniques (Table 1).

One of the concepts that is frequently used is the notion of frequency, which gives an indication about the dynamics of a signal. By looking at the frequency content of a signal, we can make conclusions on how fast (slow) the signal changes over time. Information about the frequency content of a signal can be deduced from its frequency spectrum, which can be computed using a mathematical transformation (Challis and Kitney, 1991). Some signals change very rapidly, and are therefore mainly associated with high-frequency components. Other signals change very slowly; the frequency spectrum of such signal is mainly comprised of a very narrow band of low frequency components. Examples of the former and the latter include EEG and cerebral hemodynamics, measured by NIRS, respectively. In addition, one can also describe the biological oscillations at different time scales via a time-frequency representation. The extraction of the oscillatory components is not only useful to reduce the complexity of the system, but can also be used to describe the underlying physiology. In the world of neurovascular coupling, the brain heart interaction takes place at long-term scale (low frequency activity), since the NIRS reflects the cardiovascular oscillations which develop in a timeframe of seconds. The speed of fluctuations of NIRS is then much lower compared to EEG oscillations. In addition, however, a NIRS signal can also contain frequency components which are faster, which are caused interference from cardiac pulsations. These frequency components are typically not considered in EEG-NIRS neurovascular coupling studies.

In addition, the recent literature on EEG-NIRS integration also focuses on the low-frequency activity of the EEG, i.e., the delta oscillations. According to Knyazev, the delta oscillation belongs to the “old brain,” which phylogenetically traces back to lizards (Knyazev, 2012). This explains why delta brushes dominate the preterm and the neonatal EEG activity. During the development, the scalp electrical activity tends to be discontinuous and is centered around a frequency between 1 and 2 Hz, which defines the periodicity of the bursts in the cortical trace (Andre et al., 2010). Consequently, delta oscillations are of primary importance in the preterm brain. An additional reason is the implication that delta oscillations regulate basic homeostatic needs, such as the blood flow circulation and normotension enforcement. The slow-wave EEG is considered as the expression of the regulation of the brainstem (which is in charge of cerebral hemodynamics) or, at least, as a projection of the subcortical activity to the cortical areas.

Finally, when comparing biomedical signals of different origin, it is very important that the time scales of the various signals are synchronized. In the setting of neurovascular coupling, this means that a rapidly changing neuronal signal (EEG) has to be matched with a very slowly varying cerebral hemodynamics signal (NIRS). In general, there are multiple approaches to overcome this problem. However, it is common practice to use a running estimate of the power of the slow wave activity, i.e., the delta oscillations. There are mainly three methods to extract such a running measure from the EEG. The most commonly used methodology is the use of the wavelet transform and average the power in the frequency band of interest, as reported by Clemenson and Lancaster (2016). A second option is to extract the delta band from an estimate of the power spectral density that is computed in running windows (Faes et al., 2015). The last option, which is very simple, but also very effective, is the use of the running root mean square (RMS) value, where the window length is chosen large enough to cut off the high frequency components, as reported by Caicedo et al. (2016).

### Methodologies to Assess Coupling

Despite the fact that research on bedside markers of neurovascular coupling is a very new domain, there are some studies available that have investigated how different signal processing techniques can be used to quantify the neurovascular coupling based on spontaneous cerebral and vascular activities. As mentioned before, these studies mainly focus on the quantification of interaction between EEG and NIRS as surrogates for brain function and brain hemodynamics, respectively.

In signal processing terms, this interaction can be regarded as a pairwise similarity, which denotes the degree to which a given time series resembles another one. Therefore, pairwise similarity measures can be used to evaluate a regulation mechanism such as neurovascular coupling. As mentioned before, the pairwise similarity belongs to the *functional connectivity* field, which focuses (only) on the statistical correlation among the investigated signals (Table 1).

The most commonly used linear similarity methods are the cross-correlation function (CCF) and magnitude squared coherence (MSC), which quantify the linear correlation between two signals at different lags or frequencies, respectively. In the world of neurovascular coupling, the former has been applied by Bari et al. (2011) to study the regulation mechanism in adults during a divided attention cognitive task. In summary, the CCF was able to reveal the presence of a cascade of responses, which was observed to be strongly influenced by the task performed by the patient. Govindan et al. (2016) used the MSC to obtain a measure for the degree of coupling between EEG and NIRS data in premature neonates and identified more signal interaction (higher MSC values) in infants without brain injury compared to other patients that died of brain injury during the course of the study.

A well-known extension of these linear methods is the wavelet coherence, which is a time-variant representation of the neurovascular coupling dynamics, as performed by Musizza et al. (2007) and Chalak et al. (2017). The main advantage of using a wavelet coherence approach is the fact that this technique is able to deal with the non-stationary nature of NIRS and EEG signals. Chalak et al. (2017) performed the time-frequency coherence analysis on the raw data of amplitude-integrated EEG and brain oxygenation signals, while Musizza applied the continuous wavelet transform to extract the oscillation of interest (with frequency below 0.05 Hz). In both studies, the authors emphasize the importance of phase coupling, which defines at which time delay the oscillations lock in.

A further development consists in the usage of the *effective connectivity*, which takes into account the directionality of the neurovascular coupling (Table 1). Caicedo et al. (2016) have applied the linear transfer entropy in neonates to quantify the directionality between NIRS signals and the RMS of the EEG (where the window length was chosen large enough to capture only the delta oscillations). In general, transfer entropy assesses whether the current transient of a signal can be explained by the past trajectory of another signal (Lee et al., 2012). More specifically, Caicedo et al. (2016) found that the cortical activity is caused by the hemodynamic and metabolic course of the brain. Remarkably, however, the directionality was found to be reversed when, in addition to the linear interactions, also the non-linear interactions were taken into account (Hendrikx et al., 2018a).

Another mathematical technique to study directionality is the use of parametric transfer function models, as applied by Talukdar et al. (2015). In this study, multiple sets of gamma transfer functions were used. Using such sets, the authors were able to predict NIRS hemodynamics from EEG spectral envelopes, indicating that the gamma transfer function approach can be used to study neurovascular coupling. In addition, the resulting parameters of the model could provide additional insights into the neurovascular coupling mechanism.

To the best of our knowledge, there are currently no studies available that have investigated non-linear signal processing techniques, except for the preliminary results reported by Hendrikx et al. (2018a). In addition to the methods discussed in this section, other methodologies can be considered for the computation of neurovascular coupling markers, based on studies of the brain heart connectivity literature:

(1) The first option is to assess the interaction delays among signals via the cross-correlation landscape [time delay stability (TDS)].
(2) One can extend the transfer entropy to a model-free approach, in order to capture any possible type of interaction, i.e., linear and non-linear interactions.
(3) The combination of the previous two points can be regarded as a general type of phase coupling, which looks only at the phase of the signal and their non-linear interaction.

### Graph Theory

#### Including More Signal Modalities in the Assessment of Neurovascular Coupling

In the previous section, several techniques were introduced to compute parameters for neurovascular coupling, based on multimodal processing of NIRS and EEG signals. From a broader perspective, however, there are in fact multiple regulation mechanisms that are working simultaneously in order to provide and maintain an adequate brain perfusion (Banaji et al., 2005). On a very general level, two major regulation mechanisms can be distinguished in addition to the neurovascular coupling: cerebral autoregulation and cerebral oxygen balance (Smits et al., 2017). The former ensures that cerebral blood flow is kept more or less constant, despite variations in cerebral perfusion pressure, while the latter determines the relation between oxygen delivery and metabolic demand of the brain.

A concise study on brain perfusion thus has to take into account that multiple regulation mechanisms are working at the same time. Therefore, such an analysis requires the incorporation of other signal modalities besides EEG and NIRS, such as arterial blood pressure (ABP) and fractional tissue oxygen extraction (FTOE). The former is needed to compute a marker for cerebral autoregulation, while the latter can be used to get insight into the cerebral oxygen balance (Naulaers et al., 2007). A simultaneous analysis of EEG, NIRS, ABP, and FTOE measurements leads to the definition of three markers, each indicating and assessing the status of one of the regulation mechanisms that are essential in providing the brain with a proper amount of energy in order to support its function.

Once all of these signals are available, recent studies indicate that it is also useful to compare the interaction between the remaining pairs of signals. Indeed, Semenova et al. (2017) compared the dynamics of EEG and ABP signals in preterm neonates and found that a higher degree of EEG-ABP interaction is associated with a worse clinical outcome, assessed using clinical risk index for babies II scores. Furthermore, if we add other signals like HR, which is known to have a key influence on cerebral hemodynamics in preterm infants, simultaneous markers on HR passivity can be obtained (Mitra et al., 2014). Such markers were found to be of diagnostic value in identifying impaired cerebrovascular reactivity, leading to adverse clinical outcome. In summary, it is important to incorporate a wide variety of signal modalities in order to enable an adequate study on brain perfusion, since the pairwise interactions between the different modalities can be used as physiological markers.

One of the first studies that investigated the simultaneous interactions in a multimodal dataset, is a study by Pfurtscheller et al. (2008). More specifically, in this study, NIRS, EEG, ABP, respiration and HR were combined with cross spectral and sliding cross correlation calculations, which are both linear, functional methods (Table 1). Results indicate that slow precentral (de)oxyhemoglobin concentration oscillations during awake rest can be temporarily coupled with EEG fluctuations in sensorimotor areas and modulate the excitability level in the brains’ motor areas, respectively. Therefore, this study provides support for the idea that resting state networks fluctuate with frequencies between 0.01 and 0.1 Hz (Mantini et al., 2007).

#### Graph Theory for Functional Connectivity

The main limitation of the methodology outlined above is the combinatorial number of couplings based on the variables involved. If one starts with five variables (e.g., HR, ABP, SpO_2_, NIRS, and EEG), one gets 10 couples of interaction. The complexity of the interaction problem thus increases exponentially, which is difficult to manage statistically (Pavlopoulos et al., 2011). Furthermore, it is often of interest to study the interaction among systems, rather than the interaction between single signals (e.g., the interaction between the cerebral system and the cardiovascular system). A possible solution is the description of the couplings among variables as a graph of a network, whose activity can be compactly represented using graph theory.

Formally, a graph is defined by a non-zero number of nodes (vertices) and a number of links (edges) between these nodes. In general, graphs can be used to describe a great variety of real-world situations, which explains the extensive use of these mathematical objects in many different fields. A good introduction on graph theory mathematics and an overview of the numerous applications of graph theory is presented in Pavlopoulos et al. (2011). Using graph structures has some advantages:

(1) It is a straightforward methodology to deal with the exponentially increasing number of interaction pairs when adding signals to the analysis.
(2) It allows for a clear and visual representation.
(3) It allows to study clusters of signals in a straightforward way. This is especially useful when the interaction between different subsystems is of interest, rather than the interaction between the signals themselves.
(4) The behavior of the network as a whole can easily be studied, using the extensive methods on connectivity that are available in literature.

Despite the fact that graph theory was only recently introduced in the field of Network Physiology, it has been used extensively in studies on functional connectivity of the brain, as outlined by Fallani et al. (2010). Naturally, the brain is modeled by a graph in these studies, which allows to study the interactions between different brain regions. Depending on the number of nodes, it can even be possible to study the interactions in one particular brain region. However, in such case, the number of nodes has to be large enough, which is mainly limited by the data at hand, i.e., the number of channels in the multichannel EEG recording. In general, studies on functional connectivity of the brain build graph models using one signal or imaging modality, mostly EEG (Hata et al., 2016; Tokariev et al., 2016) or fMRI (Wang et al., 2010; Smitha et al., 2017).

Gao et al. (2011) constructed graph models from fMRI BOLD data recorded in a large cohort of normal pediatric subjects. Using sequential measurements, they observed a remarkable improvement in whole brain wiring from 3 weeks up to 2 years after birth, which is a critical time period for brain development. Lavanga et al. (2018) investigated the use of graph models constructed from multichannel EEG measurements in order to construct a brain-age model for preterm neonates (Figure 3). Using 8-channel EEG measurements, they observed a decrease in functional connectivity with post-menstrual age (PMA). In addition, the functional connectivity was found to be an accurate predictor for the age of neonates.

#### Graph Theory in Network Physiology

**FIGURE 2:**
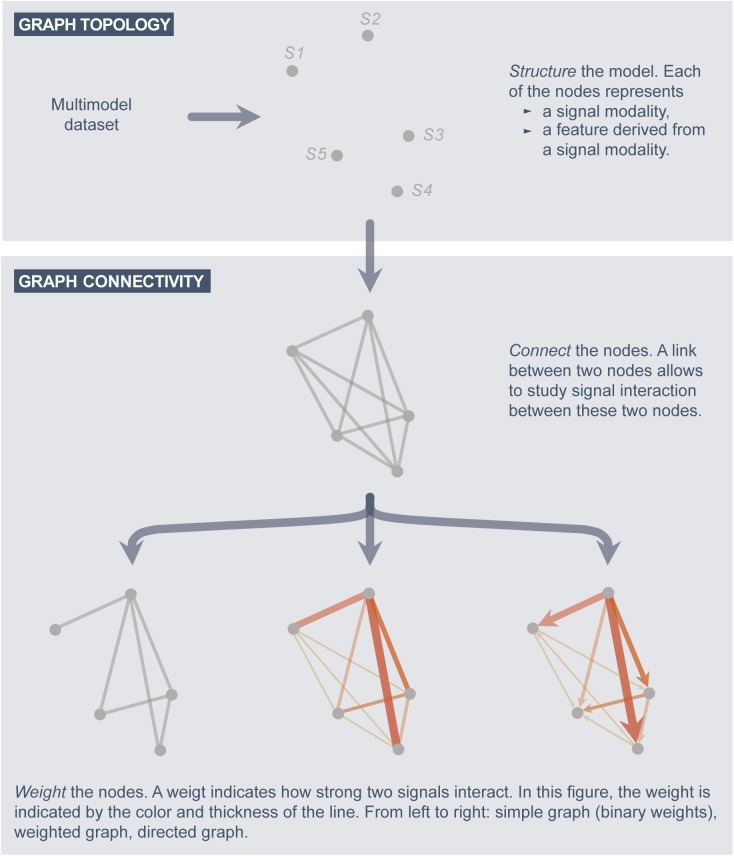
A systematic approach to construct graph models.

In the field of Network Physiology, graph models are constructed from a multimodal dataset (Bashan et al., 2012; Bartsch et al., 2015). Before constructing such graphs, one has to make sure that the time scales of the signals are synchronized, as explained in subsection G1. Afterward, a signal interaction graph is constructed as follows (Figure 2).

##### Graph topology

The structure of the graph is defined by the nodes. In a signal interaction graph, a node corresponds to one particular signal modality of the analysis, or a feature extracted from a signal (e.g., the power in the delta frequency band of the EEG).

##### Graph connectivity

(a) Define a link between each two nodes for which the signal coupling is of interest.
(b) Associate each link with a weight value, which indicates the strength of the signal interaction (coupling) and/or the directionality.

**FIGURE 3:**
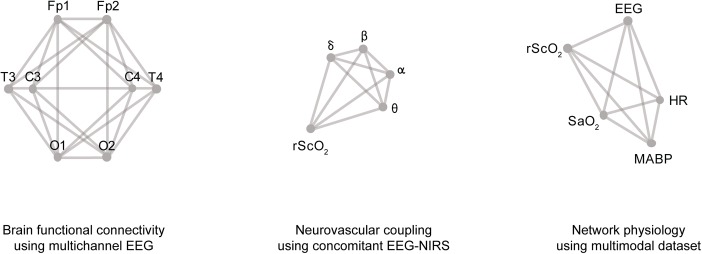
Examples of graph models in biomedical data processing, from left to right: a graph model used for functional connectivity using 8-channel EEG; a graph model to quantify neurovascular coupling by incorporating different frequency bands of the EEG and a NIRS signal; and a graph model to study network physiology, which includes five different signal modalities.

Regarding the *graph topology*, there is an endless amount of possibilities to define the structure of signal interaction graphs. Some networks to model physiological function can be quite simple (Bashan et al., 2012), while other networks are more detailed, and thus, more complex (Bartsch et al., 2015). To illustrate the variety of applications in the field of biomedical signal processing, some examples are presented in Figure 3, based on studies by Hendrikx et al. (2018b) and Lavanga et al. (2018). For each application, a graph with a specific topology can be constructed, based on the research question(s) of the analysis. The structure of the graph is limited by the signal modalities available in the dataset and/or the features derived from these signals.

Regarding the *graph connectivity*, there is again a wide variety of design choices on how to connect the nodes and how to define the weights associated with the links. First of all, one has to define which nodes are connected and which nodes lack a connection. Second, one has to specify how the weights associated with the links are computed. This is equivalent to the choice of one particular signal processing technique, since the weights denote signal interaction (coupling). Which signal processing technique to use has to be defined by the researcher, based on the characteristics of the research. In particular, one has to think about the nature of the interactions that needs to be captured: *linear* versus *non-linear* interactions and similarity (*functional* methods) versus directionality (*effective* methods). The most commonly used signal processing techniques are summarized in Table 1. In the setting of graph connectivity, two additional remarks need to be taken into account.

As a first remark, it is important to note that the graphs depicted in Figure 3 are characterized by the presence of a link between every pair of nodes. Formally, this type of graph is referred to as a *complete* graph. Such graphs are the most general type of graphs, since they allow to investigate the interaction between every possible pair of signals. It is important to note that in general it is always a good strategy to consider a link between every pair of signals, even when the signals are not expected to interact. Indeed, the weights of such links could be used as a validation for signal processing technique used to define the signal interaction: can the method detect that these signals are not interacting?

As a second remark, it is also possible to determine the presence (or absence) of a link automatically. In such an analysis, the weights are generally chosen binary. Graphs which only include the presence and absence of links (and no specific weight value), are formally referred to as *simple* graphs. An example: one could assign a ‘1’ (presence of a link) if there is a significant correlation between two signals, while a ‘0’ (absence of a link) denotes the lack of a significant correlation. In this setting, it is thus not necessary to manually define the presence of the links, since this is automatically detected by the signal processing technique.

The first study on the use of graph models in the field of Network Physiology is a study by Bashan et al. (2012). This study demonstrated that each physiological state is characterized by a specific network structure, demonstrating a robust interplay between network topology and function. Furthermore, high network flexibility in response to perturbations was observed, since the network was observed to be able to reorganize on time scales of a few minutes. A follow-up study on graph models in Network Physiology, is a study by Bartsch et al. (2015), who used the TDS framework in order to define the strength of the signal interactions. In short, TDS is based on linear correlations and is therefore only able of capturing linear signal interactions. The results of this study again demonstrated a direct association between network topology and physiologic function. In addition, the graph models were found to be useful in understanding how health and distinct physiologic states emerge from networked interactions among non-linear multi-component complex systems.

A second study on graph models in the field of Network Physiology is a study by our own group (Hendrikx et al., 2018b). In this study, graph models were used to investigate the interaction between cerebral hemodynamics, brain function and systemic variables. The graph models were constructed using the RBF kernel function, which is a non-linear similarity measure. Therefore, the methodological framework is able to capture non-linear signal interactions. The study showed that graph theory can be used to capture changes in signal interaction accurately and that the resulting graph models can be used to study the difference between distinct physiological states (Hendrikx et al., 2018b).

Graph models do not only provide a compact, efficient, and statistically well-defined framework to study simultaneous signal interaction, but they are in fact an entirely new way of thinking, which has been shown to produce an added value in multiple studies already (Bashan et al., 2012; Bartsch et al., 2015; Hendrikx et al., 2018b). The usage of interaction and coupling techniques provides an in-depth view of the interaction among physiological systems, moving from sectorial or univariate medicine to a more holistic approach. The latter is well-suited in case of continuous monitoring of critical states (e.g., in cases where it’s likely to have multiple organ failures), and the well-being and mental status of a person, which is known to rely on the interaction between multiple systems (Chaparro-Vargas et al., 2016). Moreover, the necessity to improve the sleep quality and the developmental and cognitive abilities of neonates in the near future (which interact differently in neonates compared to adults) will require the summarized view of a graph approach instead of long recordings of multiple apparently unrelated variables. In this perspective, Network Physiology gives a broader view on the body activity (Bashan et al., 2012), comparable to the broader view on the cortical status given by the brain connectivity at its discovery (Friston, 1994).

In general, the overall purpose of the signal processing techniques (such as computational graph models) is to obtain physiological markers starting from raw measurements that are routinely obtained in neonatal intensive care units. These physiological markers could in turn be used in clinical practice to make neonatal intensive care more patient-specific. In addition, signal processing advancements are also of high importance in neonatal pharmacological studies, since the effect of medication on the neurovascular coupling in neonates is yet unclear (Smits et al., 2017). Neonates are exposed to a large number of medications, most of which are used off-label in infants because clinical trials for safety, dosing, and efficacy of drugs are lacking in this population (Hsieh et al., 2014; Ku and Smith, 2015). In this setting, signal processing techniques could thus be used to identify neonates at risk for neurodevelopmental disabilities and could assist clinicians in making a timely diagnosis, which enables to start appropriate personalized therapies early. Therefore, this patient-centered neonatal intensive care can help to reduce the occurrence of neurodevelopmental disabilities in the preterm population (Molteno et al., 1999; Cioni et al., 2016).

## Conclusion

We suggest that further research in the field of physiology is required in order to gain more insight into the exact working principle of the neurovascular coupling in neonates (*spatiotemporal* studies). In addition, further studies on the use of signal processing in EEG-NIRS integration are also required (*general* studies). Such studies enable to construct new software, based on algorithms that describe the relation between cerebral metabolism and oxygen delivery. Graph models in particular can be of special interest in future studies, since these models allow to study the simultaneous action of different regulation mechanisms, which is essential in studies on brain perfusion. These new measurements will provide a better understanding of the coupling mechanisms in the neonatal brain, and will eventually lead to an improvement of the neonatal intensive care in general.

## Author Contributions

DH, ML, and GN wrote the review together. AS, ODW, LT, KJ, AC, and SVH reviewed and corrected the text.

## Conflict of Interest Statement

The authors declare that the research was conducted in the absence of any commercial or financial relationships that could be construed as a potential conflict of interest.
